# Investigating a paralytic shellfish poisoning in Gando Village, Wete District, Tanzania, July 2015

**DOI:** 10.11604/pamj.supp.2018.30.1.15265

**Published:** 2018-05-17

**Authors:** Loveness John Urio, Joseph Asamoah Frimpong, Innocent Semali, Rogath Saika Kishimba, Janneth Maridadi Mghamba, Ahmed Abade, Senga Sembuche, Nsiande Lema, Asha Khamis Ussi

**Affiliations:** 1 Tanzania Field Epidemiology and Laboratory Training Program, Tanzania; 2 African Field Epidemiology Network, Accra, Ghana; 3 Muhimbili University of Health and Allied Sciences, Tanzania

**Keywords:** Outbreak investigation, paralytic shellfish, poisoning, Tanzania

## Abstract

The investigation of foodborne outbreaks requires a multi-disciplinary set of skills. Frequently, foodborne-related outbreaks are poorly investigated due to lack of all required skills on the part of the investigators. This case study, based on a shellfish poisoning outbreak investigation conducted in Wete, Zanzibar in July 2015 by the Tanzania Field Epidemiology Training Program (TFETP), seeks to reinforce principles and skills in foodborne outbreak investigation. It is primarily intended for training public health practitioners in a classroom setting. Facilitating this case study should take approximately 3 hours.

## How to use this Study

**General instructions:** ideally, 1 to 2 instructors facilitate the case study for 10 to 20 students in a classroom or conference room. The instructor should direct participants to read a paragraph out loud, going around the room to give each participant a chance to read. When the participant reads a question, the instructor directs all participants to perform calculations, construct graphs, or engage in discussions. The instructor may split the class to play different roles or take different sides in answering a question. As a result, participants learn from each other, not just from the instructors. Specific instructor’s notes are included with each question in the instructor’s version of this case study.

**Audience:** residents in Field Epidemiology and Laboratory Training Programs (FELTPs), and others who are interested in this topic.

**Prerequisites:** before using this case study, case study participants should have received training in basic biostatistics in epidemiology, public health surveillance, and advanced epidemiology and outbreak investigation and response.

**Materials needed:** laptop with Microsoft Excel or graph paper, Epi info software, flipchart or white board with markers.

**Level of training and associated public health activity:** intermediate outbreak investigation

**Time required**: 3 hours

**Language**: English

## Competing interest

The authors declare no competing interest.
